# Are Subject-Specific Musculoskeletal Models Robust to the Uncertainties in Parameter Identification?

**DOI:** 10.1371/journal.pone.0112625

**Published:** 2014-11-12

**Authors:** Giordano Valente, Lorenzo Pitto, Debora Testi, Ajay Seth, Scott L. Delp, Rita Stagni, Marco Viceconti, Fulvia Taddei

**Affiliations:** 1 Medical Technology Laboratory, Rizzoli Orthopaedic Institute, Bologna, Italy; 2 BioComputing Competence Centre, SCS s.r.l., Bologna, Italy; 3 Department of Bioengineering, Stanford University, Stanford, California, United States of America; 4 Department of Mechanical Engineering, Stanford University, Stanford, California, United States of America; 5 Department of Electrical, Electronic and Information Engineering, University of Bologna, Bologna, Italy; 6 Department of Mechanical Engineering and INSIGNEO Institute for *In Silico* Medicine, University of Sheffield, Sheffield, United Kingdom; Politecnico di Milano, Italy

## Abstract

Subject-specific musculoskeletal modeling can be applied to study musculoskeletal disorders, allowing inclusion of personalized anatomy and properties. Independent of the tools used for model creation, there are unavoidable uncertainties associated with parameter identification, whose effect on model predictions is still not fully understood. The aim of the present study was to analyze the sensitivity of subject-specific model predictions (i.e., joint angles, joint moments, muscle and joint contact forces) during walking to the uncertainties in the identification of body landmark positions, maximum muscle tension and musculotendon geometry. To this aim, we created an MRI-based musculoskeletal model of the lower limbs, defined as a 7-segment, 10-degree-of-freedom articulated linkage, actuated by 84 musculotendon units. We then performed a Monte-Carlo probabilistic analysis perturbing model parameters according to their uncertainty, and solving a typical inverse dynamics and static optimization problem using 500 models that included the different sets of perturbed variable values. Model creation and gait simulations were performed by using freely available software that we developed to standardize the process of model creation, integrate with OpenSim and create probabilistic simulations of movement. The uncertainties in input variables had a moderate effect on model predictions, as muscle and joint contact forces showed maximum standard deviation of 0.3 times body-weight and maximum range of 2.1 times body-weight. In addition, the output variables significantly correlated with few input variables (up to 7 out of 312) across the gait cycle, including the geometry definition of larger muscles and the maximum muscle tension in limited gait portions. Although we found subject-specific models not markedly sensitive to parameter identification, researchers should be aware of the model precision in relation to the intended application. In fact, force predictions could be affected by an uncertainty in the same order of magnitude of its value, although this condition has low probability to occur.

## Introduction

Advances in computing power and numerical methods for modeling and simulation of movement are expanding the use of computational models of the musculoskeletal system in research and clinical applications [1], [2]. Calculation of muscle and joint forces represent a challenging modeling application [3], [4]. Because musculoskeletal geometry and tissue properties can vary markedly among individuals, the accuracy of generic models has been questioned [5], [6], particularly when studying musculoskeletal disorders [7], [8]. Conversely, subject-specific models allow inclusion of individual musculoskeletal anatomy and properties, providing an alternative approach to calculating muscle moment arms [9], [10], muscle and joint forces [11], [12], bone and cartilage stresses [13], [14].

In general, analyses of musculoskeletal dynamics require the use of musculoskeletal models and the application of rigid body dynamics and optimization methods to calculate muscle forces [2], [15]. Until now, the creation of subject-specific musculoskeletal models and simulations of movement has represented a time-consuming process, and there has been limited modeling software available to standardize the process and make musculoskeletal modeling more efficient. Consequently, few attempts have been made to create subject-specific models and study musculoskeletal pathological conditions (e.g., [16]–[18]). In fact, model creation requires data collections from different technology (e.g., MRI, gait analysis), and processing the data to create a model of musculoskeletal dynamics. The process involves the definition and calculation of subject-specific modeling parameters from imaging data, including the identification of: tissue volumes and densities to calculate body inertial properties; body landmark positions to define joint reference frames and constraints; muscle attachment points to define the geometry of muscles; and muscle architecture parameters to calculate muscle force-generating capacities.

Independent of the software used, there are unavoidable uncertainties in parameter identification during the process of model creation. These uncertainties have different sources: they can be operator-dependent (e.g., when a user identifies body landmark positions and point positions of musculotendon actuators), or related to the unavailability of measurements *in vivo*, such as maximum muscle tension and musculotendon architecture parameters (e.g., muscle physiological cross-sectional area, fiber length and tendon slack length). Sensitivity analyses to different parameters have been performed to assess variations in model predictions and determine which parameters have the most influence (e.g., [19]–[21]). However, these analyses have not assessed how the uncertainties associated with the creation of subject-specific musculoskeletal models, and their combined effect, may affect model predictions.

Therefore, the aim of the present study is to analyze the sensitivity of subject-specific model predictions (i.e., joint angles, joint moments, muscle and joint contact forces) during walking to the uncertainties in the values for model parameters. To achieve this aim, we first created a musculoskeletal model of the lower limbs from MRI of a healthy subject. We then performed a Monte-Carlo probabilistic analysis accounting for the uncertainties associated with the creation of the model, including body landmark positions, maximum muscle tension and musculotendon geometry. The analysis was performed by using freely available musculoskeletal modeling software that we developed in an effort to standardize subject-specific model creation and generate accurate models using an efficient workflow. The modeling software integrates with OpenSim [22], a widely used multibody-dynamics solver adopted in musculoskeletal applications (e.g., [19], [23], [24]).

## Materials and Methods

### Ethics statement

This study was approved by the Bioethical Committee of the University of Bologna, Italy (July 7, 2012). Written informed consent was obtained from the participant.

### Experimental data

One healthy subject (male; age: 31 years; height: 183 cm; weight: 70.5 kg) volunteered to participate in this study. The experimental data collection included lower-body MRI scans and gait analysis data, freely available at the dedicated SimTK.org project page (https://simtk.org) and described as follows.

Pelvis and lower limbs were imaged using a 1.5 T MR scanner (Intera, Koninklijke Philips N.V., The Netherlands). Four series of images were obtained at different resolutions: a full lower-body scan (T1-weighted Magnetization Transfer, 5 mm slice thickness, 5.5 mm slice spacing, resolution of 512×512 pixels), and three higher resolution acquisitions at the hip (T1-weighted High Resolution Turbo Spin Echo, 5 mm slice thickness, 5.5 mm slice spacing, resolution of 864×864 pixels), at the knee (T1-weighted Turbo Spin Echo, 3 mm slice thickness, 3.3 mm slice spacing, resolution of 560×560 pixels) and at the ankle (T1-weighted Turbo Spin Echo, 3 mm slice thickness, 3.3 mm slice spacing, resolution of 1024×1024 pixels) joint regions.

The subject was assessed by means of gait analysis. The experiment was carried out using a stereophotogrammetric system (SMART-D BTS, Milano, Italy) and two force platforms (Bertec Corporation, USA). Twenty-nine retro-reflective markers were attached to the pelvis, thighs, shanks and feet of the analyzed subject. A trial of level walking at self-selected speed was carried out. Joint neutral position was collected from a standing posture, as well as joint flexion position from a seated posture. All data were collected at 200 samples per second. Relevant anatomical landmarks [25] were calibrated in standing and flexed posture using the pointer technique illustrated in Cappozzo et al. [26]. Segmental kinematics of the pelvis and lower limbs was reconstructed via a C.A.S.T. approach [26] with double calibration [27] to minimize soft tissue artifact propagation.

### Workflow of subject-specific musculoskeletal modeling

**Figure 1:**
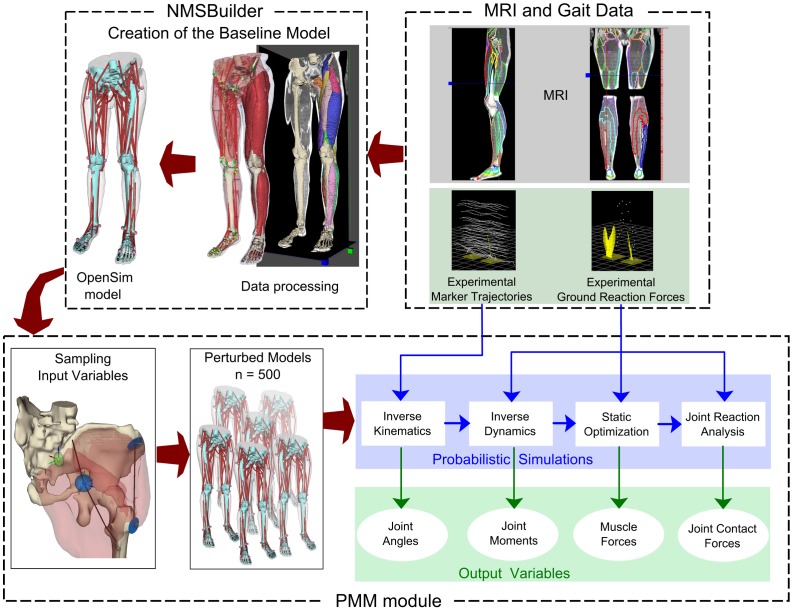
Workflow of subject-specific musculoskeletal modeling. The modeling software systems were applied to study the sensitivity of model predictions to the uncertainties in parameter identification. Lower-body MRI and gait analysis data were acquired for a healthy subject. NMSBuilder was used to create the baseline subject-specific model leveraging OpenSim. The Probabilistic Musculoskeletal Modeling module (PMM) was used to create probabilistic simulations of gait through a Monte-Carlo analysis, by interfacing Matlab and OpenSim. The input variables were perturbed according to their uncertainties, and the corresponding OpenSim models were created that included the different sets of perturbed variables. Using each model and the recorded gait analysis data, simulations of gait were run to calculate the stochastic output variables.

We investigated the robustness of model predictions to the uncertainties in the identification of the parameters needed to create an image-based musculoskeletal model of the lower limbs, using MRI and gait data (Figure 1). To the purpose, freely available software that we developed, i.e., NMSBuilder and the Probabilistic Musculoskeletal Modeling module (PMM), was used to create the baseline subject-specific model and perform probabilistic simulations of gait, leveraging OpenSim. Additional details on the software system can be found in the Appendix S1. All of the software is available at the dedicated SimTK.org project page (https://simtk.org).

### Baseline subject-specific model

The model used in this study was defined as a 7-segment, 10-degree-of-freedom (DOF) articulated system, actuated by 84 musculotendon units, and referred to as the baseline model. The seven rigid bodies included pelvis, thighs, shanks and feet. Each body volume was derived from the MR images, and the inertial properties (mass, center of mass and moments of inertia) were calculated assuming each body composed of two parts, the bone and soft tissue, having uniform densities of 1.42 g/cm^3^ and 1.03 g/cm^3^ [28], respectively. Each hip was modeled as a 3 DOF ball-and-socket joint, each knee and ankle as a 1 DOF hinge joint. Body and joint coordinate systems were identified according to the ISB standards [29]. The hip joint was defined at the center of the femoral head, the knee axis of rotation was defined as the trans-epcondylar line [30], and the ankle axis of rotation was defined as the trans-malleolar line [31]. The number and paths of the musculotendon actuators were defined according to the generic model proposed by Delp and co-workers [32]. The model includes one or more lines of action per muscle, acting between origin points on the proximal body and insertion points on the distal body. Intermediate via-points are included to model the paths of muscles wrapping over underlying structures. The maximum isometric force (*F_max_*) of each musculotendon unit (*i*) was estimated, assuming muscle fiber length proportional to musculotendon length [33], as: 
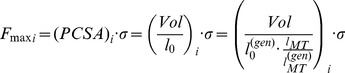
(1) where *PCSA* is the muscle physiological cross-sectional area, *Vol* is the muscle volume calculated from MRI, 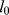 and 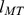 are the optimal fiber length (unknown) and the musculotendon length (calculated from MRI) for the subject-specific model, respectively, 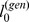 and 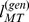 are the corresponding quantities for the generic model [32], and *σ* is the maximum muscle tension set to 61 N/cm^2^ [34].

To create the baseline model, bone and soft tissue meshes (pelvis, thighs, shanks and feet) were segmented semi-automatically using Amira (Visage Imaging, Berlin, Germany). NMSBuilder was then used to create the subject-specific musculoskeletal model. The segmented surfaces were imported in NMSBuilder as STL files, and were divided into seven body districts, each made of bone and soft tissue parts [35]. The data were organized into a hierarchical structure. Different density values were then assigned to each part as metadata attributes, to calculate the inertial properties of each body. The necessary anatomical landmarks were virtually palpated [36] on the body surfaces with the help of the superimposed MR images. Subsequently, the landmark positions were used to define the reference frames of each body and the joint positions and orientations (in the parent and child bodies). The positions of musculotendon origin, via and insertion points were assigned as close as possible to those in the generic model [32]. This was done by applying an affine registration based on the body landmarks to initialize the musculotendon point positions, and then adjusting the points according to a centroid approach [37] and visually comparing their positions in the MR images. Next, the values of maximum isometric muscle force were assigned to each musculotendon unit as metadata attributes. Finally, the C++ commands of the OpenSim application programming interface (API) were generated and compiled to create the baseline OpenSim model.

### Probabilistic simulations of gait

**Figure 2:**
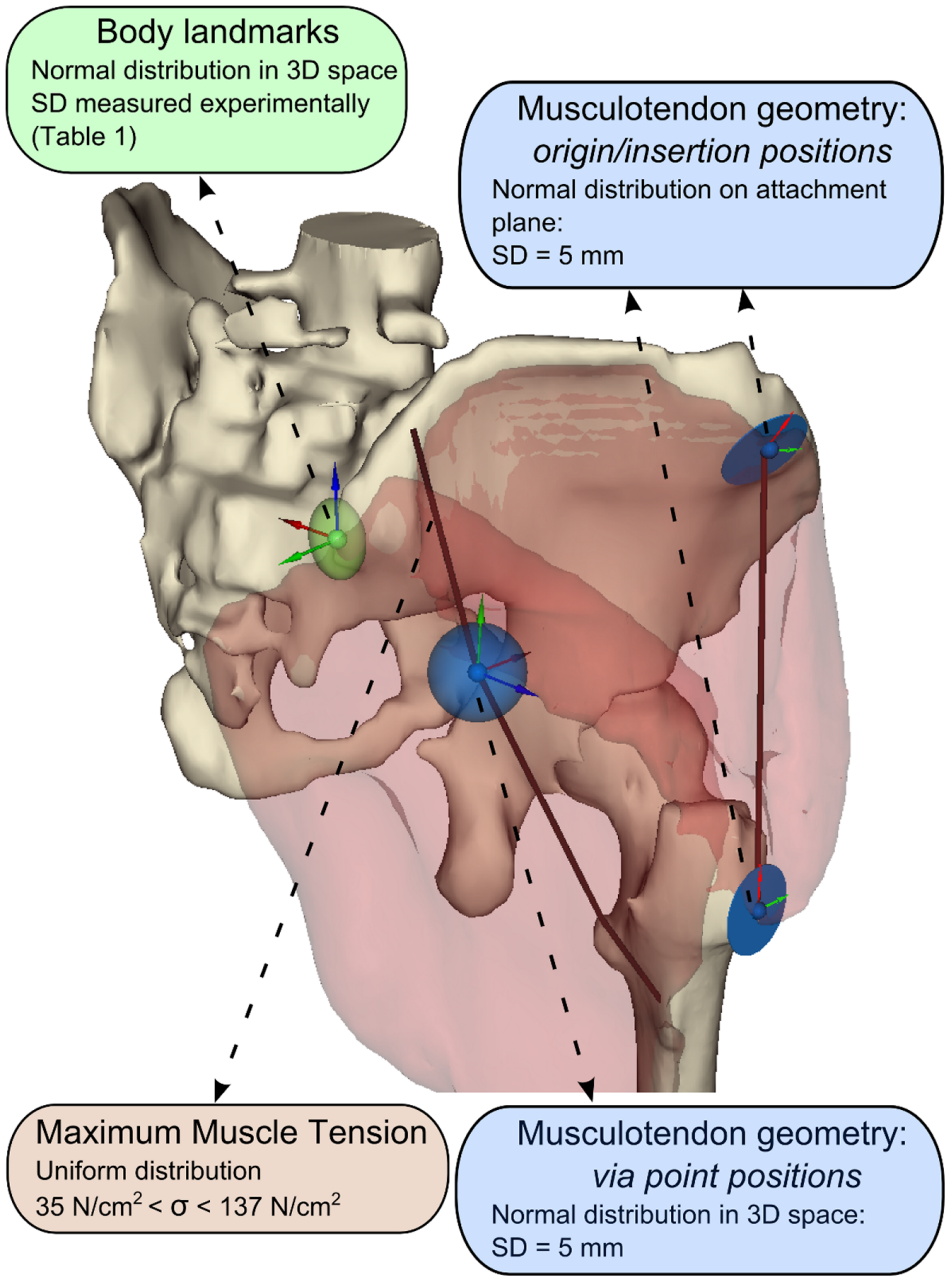
Schematic of statistical perturbation of the input variables. To analyze the sensitivity of model predictions to the uncertainties in parameter values, three categories of stochastic input variables were identified (for a total of 312 input variables): body landmark positions (affecting position and orientation of body reference frames and joints, inertial tensors and joint kinematics), musculotendon geometry (position of origin/insertion and via points defining musculotendon paths and affecting muscle moment arms) and maximum muscle tension (affecting maximum force-generating capacity of the muscles). Each variable was assumed as normally or uniformly distributed, and a Latin Hypercube Sampling strategy was applied to efficiently sample the variables from their distribution.

A probabilistic study was performed to analyze the sensitivity of model predictions to the uncertainties associated with the creation of the baseline model, given the specific articulated linkage actuated by musculotendon units represented by line segments. Therefore, three categories of variable parameters were defined (Figure 2), resulting in a total of 312 stochastic input variables:

#### 1. Body landmark positions

The x-, y- and z-coordinates of the 21 landmarks in each corresponding body reference frame were assumed as normally distributed variables. The standard deviations of each variable (Table 1) were calculated via an experimental study. In this experiment, five expert modelers used NMSBuilder to virtually palpate the landmarks on the bone surfaces three times within a time interval of two weeks. Landmark positions affect calculation of body reference frames, inertial tensors, joint positions and orientations, and joint kinematics.

**Table 1:** Standard deviations of the body landmark positions measured experimentally.

		Standard Deviation (mm)		
		X	Y	Z
**Body landmarks**	SACRUM	0.7	0.6	1.8
	RASIS	1.6	0.4	2.6
	RPSIS	0.8	0.3	2.1
	LASIS	1.2	0.6	2.3
	LPSIS	0.9	0.4	2.8
	RGT	1.0	1.4	1.1
	RME	0.4	0.7	1.3
	RLE	0.6	1.6	1.3
	RHC	0.6	0.8	1.5
	RHF	2.2	0.8	0.3
	RTT	3.5	1.3	4.2
	RLC	0.7	3.5	1.2
	RMC	0.5	1.5	0.6
	RMM	1.6	0.9	0.5
	RLM	0.7	0.5	0.3
	RCA	1.1	1.0	0.3
	RFM	0.8	1.6	0.1
	RSM	0.8	0.7	1.0
	RVM	0.7	0.7	0.4
	RPAI	0.6	1.4	0.1
	RPAII	0.6	0.5	0.0

Values were measured through virtual palpation using NMSBuilder by 5 operators in 3 trials each. X, Y and Z indicate antero-posterior, cranio-caudal and medio-lateral directions of the body reference frames, respectively. Body landmark acronyms indicate: sacrum (SACRUM), right anterior superior iliac spine (RASIS), right posterior superior iliac spine (RPSIS), left anterior superior iliac spine (LASIS), left posterior superior iliac spine (LPSIS), right great trochanter (RGT), right medial epicondyle (RME), right lateral epicondyle (RLE), right hip center (RHC), right head of fibula (RHF), right tibial tuberosity (RTT), right lateral tibial condyle (RLC), right medial tibial condyle (RMC), right medial malleolus (RMM), right lateral malleolus (RLM), right calcaneus (RCA), right first metatarsus (RFM), right second metatarsus (RSM), right fifth metatarsus (RVM), right superior plantar aspect of calcaneus (RPAI), right inferior plantar aspect of calcaneus (RPAII).

#### 2. Musculotendon geometry

The positions of the 89 points of the musculotendon paths affecting moment arm lengths were assumed as normally distributed variables. The points included origins, pseudo-origins (most distal via point on the proximal body), pseudo-insertions (most proximal via point on the distal body), and insertions, according to the definition of the different musculotendon paths. A plane approximating each musculotendon attachment area was calculated, so that each origin and insertion point position could be perturbed along two directions on the plane. Points belonging to attachment areas with large length/width ratio were approximated by a line and perturbed along one direction only. Conversely, each position of pseudo-origin and pseudo-insertion points was perturbed along the three directions of the body reference frame. Therefore, a total of 209 normally distributed variables were defined. Mean values were assumed those of the baseline model and standard deviations were set to 5 mm, as derived from the error in locating muscle attachment points from the measurement of surface landmarks [38].

#### 3. Maximum muscle tension

The maximum muscle tension (*σ*) was assumed as a uniformly distributed variable, ranging from 35 N/cm^2^ to 137 N/cm^2^ [39]. Consequently, the maximum isometric force of each musculotendon unit was calculated, using equation (1), as:
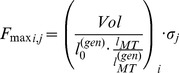
(2) where *i* is the musculotendon unit and *j* the sample of muscle specific tension within the specified range.

Uncertainties introduced by volume segmentation were not included, being segmentation a time-consuming process and hence performed by a single operator. The stance phase of one gait cycle was selected to be included in the analysis, as it is the most interesting phase from the musculoskeletal loading standpoint. PMM allowed us to perform a Monte-Carlo analysis that included kinematic and dynamic simulations of the stance phase of gait (Figure 1), leveraging the OpenSim API. The baseline model was opened in PMM, and a Latin Hypercube Sampling (LHS) strategy [40], [19] was applied to generate an efficient sampling of the input variables from their distribution. This made possible the generation of OpenSim models that included the different sets of perturbed variable values. Using each model, Inverse Kinematics, Inverse Dynamics, Static Optimization (minimizing the sum of muscle activations squared and neglecting the force-length-velocity relationships of muscle [41]) and Joint Reaction analysis were run to calculate the following stochastic output variables: joint angles, joint moments, muscle forces and joint contact forces. A convergence criterion was defined as a stopping rule for the Monte-Carlo simulations. Five-hundred simulations ensured that the output variables reached convergence. Specifically, over the last 10% of the simulations, the means and standard deviations of each output variable were within the 2% of each final mean and standard deviation [11], [19], [20]. A perturbed simulation was considered unsuccessful if joint dynamic equilibrium could not be achieved. Specifically, unsuccessful simulations occurred if the use of reserve actuators on any joint DOF exceeded 5% of the peak joint moment [24] in at least one frame of the stance phase. Preliminary analysis of the results showed that the 0.8% of the simulations run was unsuccessful, suggesting that muscle forces were generally able to generate the required joint moments. The unsuccessful simulations were excluded from the subsequent data analysis.

### Data analysis

The analysis was focused on joint angles, joint moments, major muscle forces, i.e. gluteus medius anterior (*GMedA*), middle (*GMedM*) and posterior (*GMedP*), gluteus maximus anterior (*GMaxA*), tensor fascia latae (*TFL*), *psoas*, *iliacus*, semimbranosus (*Semimem*), rectus femoris (*Rec Fem*), vastus medialis (*Vas Med*), lateralis (*Vas Lat*) and intermedius (*Vas Int*), medial (*Med Gas*) and lateral (*Lat Gas*) gastrocnemius, *soleus*, tibialis anterior (*Tib Ant*), and joint contact forces, i.e. hip, knee and ankle force magnitude. First, all quantities were expressed in percentage of the stance phase, and the force values were normalized to the subject body-weight and thus expressed in multiples of body-weight (BW). The data were then post-processed to evaluate the statistical variability in the output variables and the correlations between output and input variables. The variability was analyzed as maximum and mean standard deviation (among the output samples at each time step), and range (difference between maximum and minimum values at each time step) during the stance phase of gait. A correlation analysis was performed that evaluated the statistically significant (p<0.001) coefficients of determination (R^2^) between all output and input variables.

## Results

**Figure 3:**
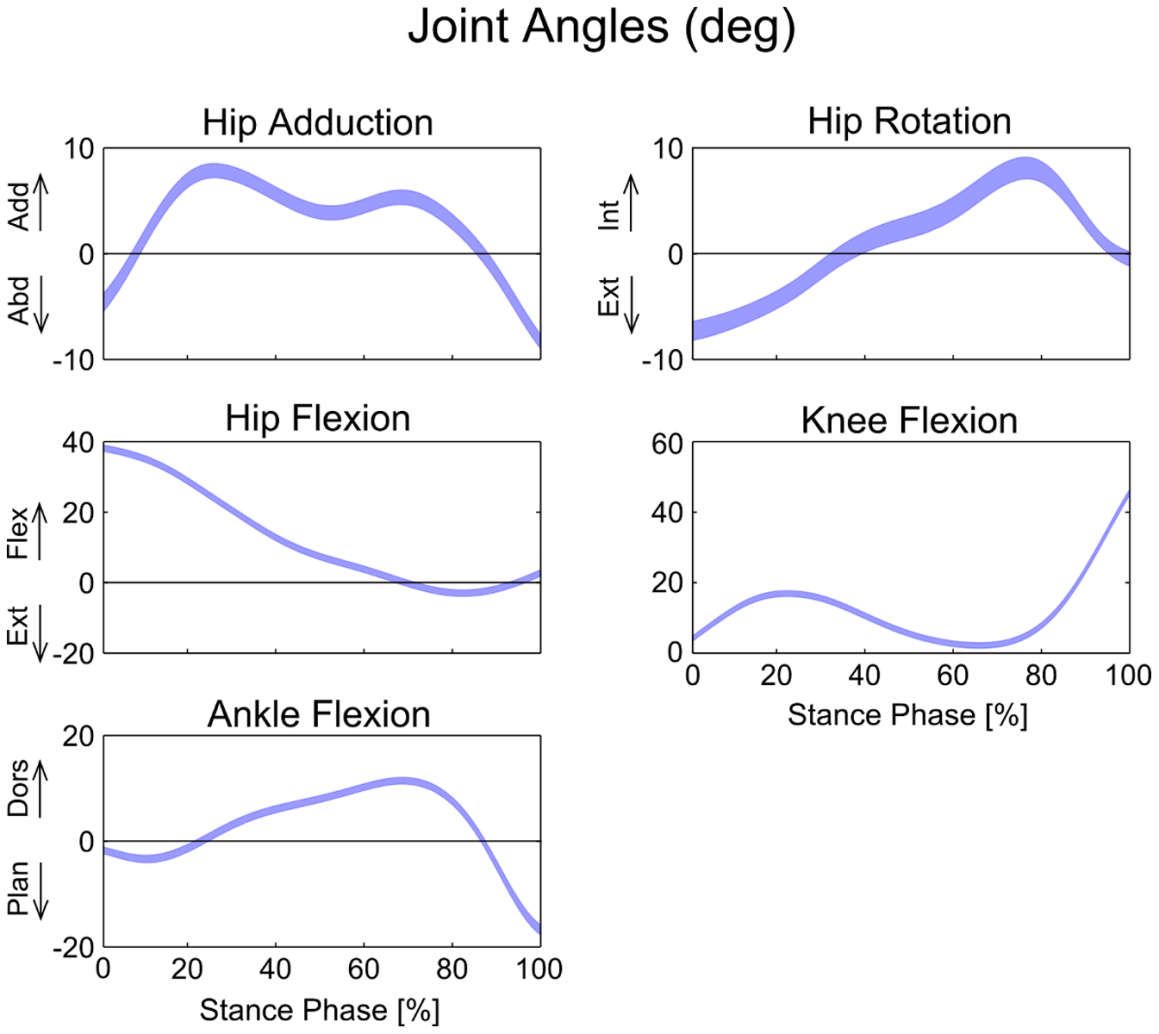
Variability in joint angles due to the perturbation of model variables. Bands represent mean values ±1 standard deviation (in degrees) during the stance phase of gait.

**Figure 4:**
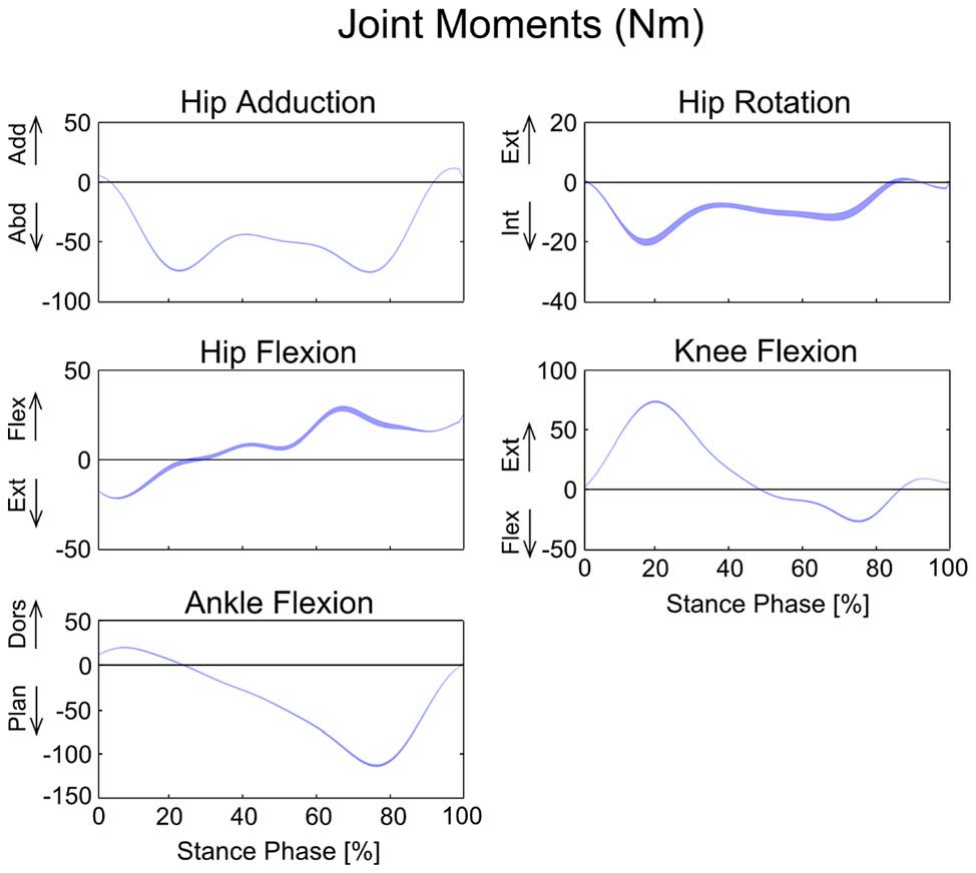
Variability in net joint moments due to the perturbation of model variables. Bands represent mean values ±1 standard deviation (in Nm) during the stance phase of gait.

**Figure 5:**
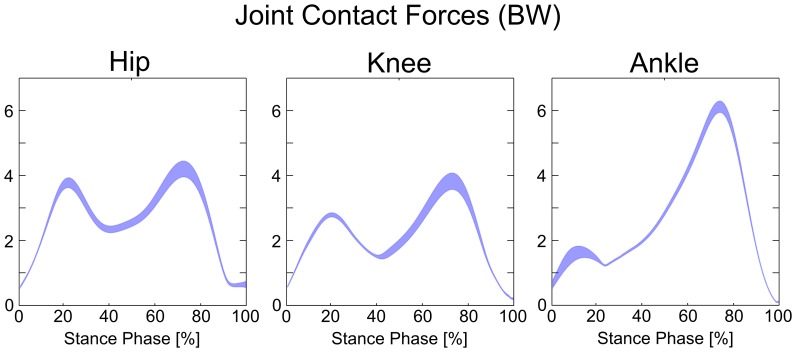
Variability in joint contact forces due to the perturbation of model variables. Bands represent mean values ±1 standard deviation (in BW) during the stance phase of gait.

**Figure 6:**
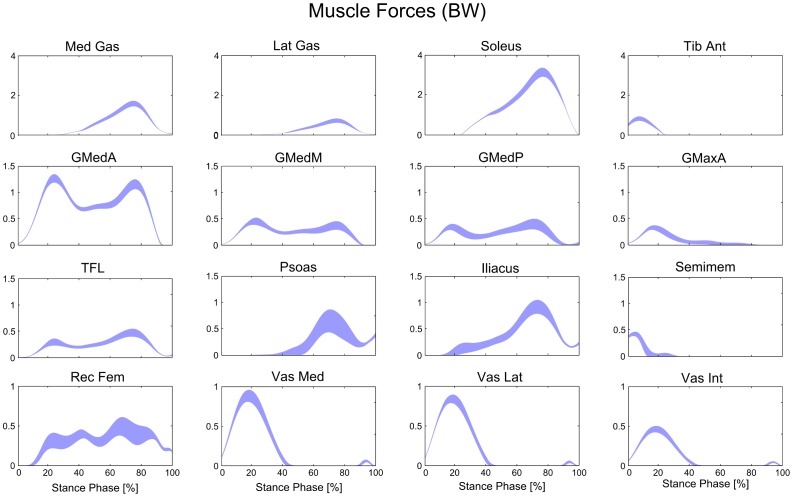
Variability in the major muscle forces due to the perturbation of model variables. Bands represent mean values ±1 standard deviation (in BW) during the stance phase of gait. Muscles shown are: medial (*Med Gas*) and lateral (*Lat Gas*) gastrocnemius, soleus, tibialis anterior (*Tib Ant*), gluteus medius anterior (*GMedA*), middle (*GMedM*) and posterior (*GMedP*), gluteus maximus anterior (*GMaxA*), tensor fascia latae (*TFL*), psoas, iliacus, semimembranosus (*Semimem*), rectus femoris (*Rec Fem*), vastus medialis (*Vas Med*), lateralis (Vas Lat) and intermedius (*Vas Int*).

The joint angles and joint moments were relatively insensitive to the expected variation in musculoskeletal parameters. We found that the maximum standard deviation among joint angles during the stance phase of gait was only 1°, and the maximum range was 7° (Figure 3). Similarly, the maximum standard deviation among joint moments from perturbation of model parameters was only 1.4 Nm, and the maximum range was 9.1 Nm (Figure 4). Joint contact forces and muscle forces presented a more marked variability compared to joint angles and joint moments. Joint contact forces showed a maximum standard deviation of 0.26 BW and a maximum range of 2.14 BW at the knee (Figure 5, Table 2). Although the standard deviations of joint contact forces were 10 times smaller than the corresponding force values, the maximum ranges presented the same order of magnitude. Muscle forces showed larger variability in *Soleus*, *Med Gas*, *Rec Fem* and *Psoas* (Figure 6, Table 2), resulting in a maximum standard deviation of 0.23 BW and a maximum range of 1.54 BW in *Soleus*.

**Table 2:** Variability in joint contact and muscle forces.

		Standard Deviation (BW)		Range (BW)	
		Mean	Max	Mean	Max
**Joint Contact Forces**	Hip	0.13	0.25	0.75	1.51
	Knee	0.11	0.26	0.84	2.14
	Ankle	0.10	0.23	0.62	1.58
**Muscle Forces**	Med Gas	0.05	0.14	0.33	0.95
	Lat Gas	0.03	0.10	0.21	0.67
	Soleus	0.08	0.23	0.54	1.54
	Tib Ant	0.02	0.10	0.11	0.68
	GMedA	0.05	0.09	0.32	0.66
	GMedM	0.04	0.08	0.25	0.51
	GmedP	0.05	0.10	0.31	0.59
	GMaxA	0.03	0.06	0.17	0.40
	TFL	0.01	0.03	0.08	0.17
	Psoas	0.05	0.15	0.33	0.89
	Iliacus	0.07	0.13	0.42	0.79
	Semimem	0.01	0.03	0.04	0.19
	Rec Fem	0.07	0.14	0.44	0.88
	Vas Med	0.01	0.04	0.07	0.24
	Vas Lat	0.02	0.07	0.11	0.35
	Vas Int	0.01	0.02	0.04	0.13

Standard deviations and ranges of the magnitudes of joint contact forces and the major muscle forces are reported as mean and maximum values across the stance phase of gait.

**Figure 7:**
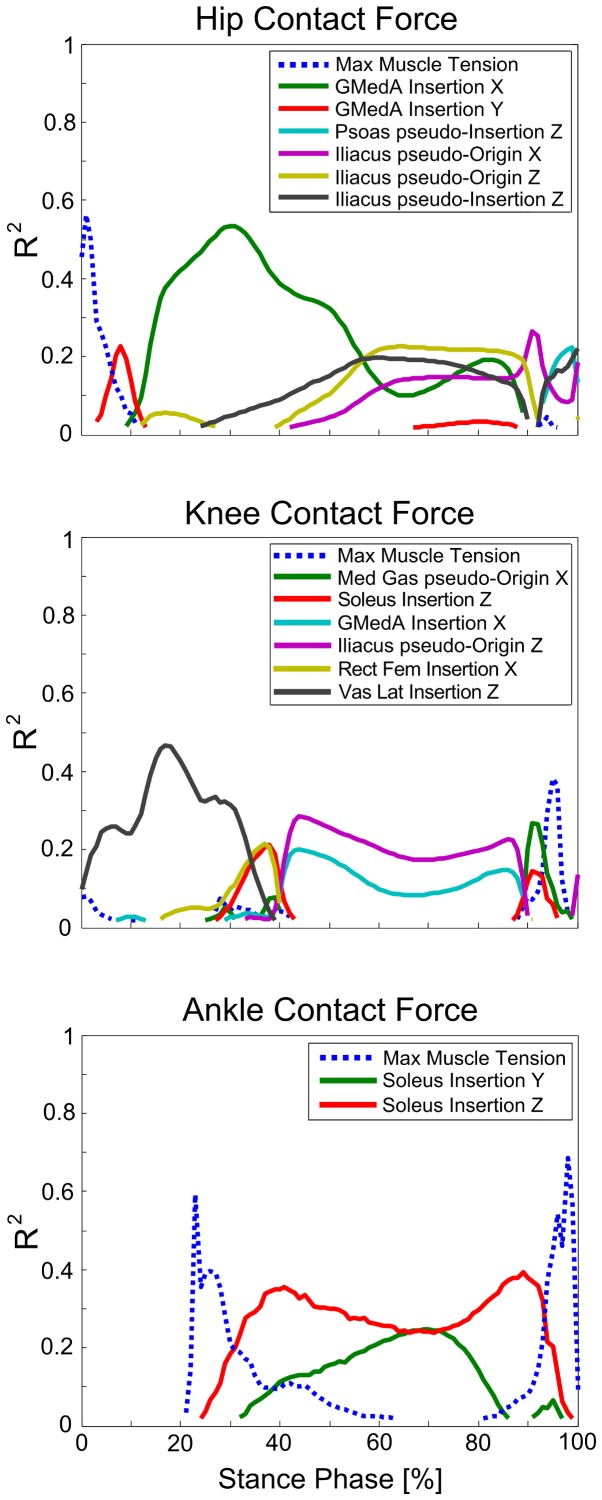
Significant R^2^ between joint contact forces and input variables during the stance phase of gait. Correlations between hip, knee and ankle joint contact forces and input variables: only statistically significant (p<0.001) R^2^ exceeding 0.2 at least in one frame during the stance phase of gait are plotted.

Given the relatively small variability in joint kinematics and kinetics, we analyzed only the correlations between joint contact forces and input variables during the stance phase of gait. Among these correlations, only 6.3% showed significant R^2^ (p<0.001). In addition, 1.3% showed significant R^2^ greater than 0.2 and never exceeding 0.7, where only seven input variables out of 312 were involved (Figure 7). The hip contact force mostly correlated with the point positions defining the geometry of *GMedA*, *Iliacus* and *Psoas*, and with the maximum muscle tension in the early stance phase. The knee contact force mostly correlated with the geometric definition of *Vas Lat*, *Iliacus* and *GMedA*, and with that of *Med Gas*, *Rec Fem* and *Soleus* for a less extended portion of stance phase. The ankle contact force mostly correlated with the geometric definition of *Soleus* and with the maximum muscle tension for a less extended portion of stance phase. The significant R^2^ between joint contact forces and body landmark positions were all less than 0.1 during the stance phase. These results (Figure 7) showed a weak correlation between output and input variables, without a marked influence of specific input variables. The sampled input variables and the complete set of post-processed output variables are available at the dedicated SimTK.org project page (https://simtk.org).

## Discussion

In this study, we analyzed the sensitivity of the predictions of an MRI-based musculoskeletal model (i.e., joint angles, joint moments, muscle and joint contact forces) during walking to the unavoidable uncertainties in parameter identification, i.e., body landmark positions, maximum muscle tension and musculotendon geometry (Figure 1).

Overall, the unavoidable uncertainties in parameter identification during the process of model creation have a moderate effect on model predictions during gait. In fact, we found that the main outcomes of model predictions, i.e., joint contact forces and muscle forces, had a maximum standard deviation of 0.26 BW across the stance phase of gait (Figure 5 and 6, Table 2). In addition, there were no critical parameters that markedly affected model predictions. We performed a correlation analysis between joint contact forces and input variables (Figure 7), and found few significant R^2^, whose values never exceeded 0.7. The input variables involved were the point positions defining the geometry of few musculotendon actuators that presented larger force-generating capacities and the maximum muscle tension in limited portions of the stance phase.

Although we found that subject-specific models are not markedly sensitive to the uncertainties in parameter identification, there is no conclusive answer to the robustness of subject-specific models. In fact, the precision of model predictions should be evaluated with regards to specific applications. For example, we found ranges (differences between maximum and minimum of the predicted value) that reached 2.1 BW in joint contact forces at the knee during the gait cycle (Table 2). In this case, the result could be affected by an uncertainty in the same order of magnitude of its value, although this condition has low probability to occur. Therefore, one should be aware of the uncertainty in musculoskeletal force predictions according to their intended application (e.g., investigation of risk of bone fracture and bone stress distribution).

To our knowledge, this is the first study investigating how the combined effect of the uncertainties in model parameters affects the predictions of a subject-specific musculoskeletal model, using a probabilistic approach. Therefore, this represents the most extended sensitivity analysis of musculoskeletal modeling predictions, providing an overall scenario of robustness of subject-specific musculoskeletal models to the uncertainties in parameter identification. Consequently, only partial or indirect comparisons with the literature were possible. We found an effect of anatomical landmark positions on predicted joint moments weaker than that showed in a previous probabilistic study limited to inverse dynamics results [21]. The uncertainties that we assigned to the landmark positions (Table 1) were lower than those in the prior study (i.e., standard deviations of 2 mm for all landmarks in each direction). We evaluated experimentally the standard deviations of the distribution by using an accurate method for landmark virtual palpation [36] implemented in NMSBuilder, which allowed us to improve the uncertainty in the identification of landmark positions. Similarly, we found a weaker effect of musculotendon geometry on predicted muscle forces compared to a previous study [42] that used a fixed-size perturbation (±10 mm) applied to each musculotendon point position along each direction of the local reference frames. Differently from that study, we assigned an uncertainty (standard deviation of 5 mm) derived from the range of landmark location errors [38], we adopted a probabilistic approach to analyze all possible configurations of musculotendon point positions, and we constrained the muscle attachment points to vary on the bone surfaces. In addition, our results generally confirm the weak influence of maximum muscle tension on the calculated muscle forces, when minimizing a cost function in static optimization problems [20], [43], [44]. We additionally found that the maximum muscle tension played a more relevant role on joint contact forces during transient phases of the gait cycle (Figure 7). Differently from previous studies, our approach explored the range of maximum muscle tension found in the literature [39] using a uniform distribution, rather than an arbitrary-size perturbation of a baseline value. However, the portions of stance phase showing larger correlations were not biomechanically relevant, as most muscles were inactive or exerted low forces.

The results of our study are affected by some limitations. We limited the study to a healthy subject and we investigated only the task of level walking as the most common daily activity. Model robustness might be different in pathological conditions and for other motor tasks such as sit-to-stand, stair ascent or descent. Although further investigations might extend our findings, the healthy subject included in this study can be considered representative of physiological conditions, and adding greater complexity was beyond the aim of the study. We did not include musculotendon parameters describing force-length-velocity relationships (i.e., optimal fiber length, tendon slack length and pennation angle). Changes in these parameters, and particularly in tendon slack length of some muscles, can markedly affect model dynamics predictions [20], [44]. However, measurements and corresponding uncertainties of these parameters are difficult to obtain *in vivo* and even by dissection studies [2]; in addition, the lack of implementation of musculotendon force-length-velocity relationships has a small influence on force predictions during walking [41]. Further, we did not consider the uncertainty introduced by representing the musculotendon units by deformable line segments in the model. However, our aim was to analyze the effect of the uncertainties in the parameters identifying a specific state-of-the art model, and including more accurate muscle path representation (e.g., [45]) would have introduced large computational costs and additional uncertainty not compatible with our analysis.

This study has relevant potentials within the computational biomechanics community. We assessed robustness of musculoskeletal models to the uncertainties in parameter identification using a probabilistic approach. Although in presence of the limitation regarding the impossibility to validate muscle forces, our results confirm that musculoskeletal models represent a promising tool that is heading towards clinical applicability, particularly to improve treatment of orthopaedic and neurological diseases [1], [15]. The analysis has been facilitated by the use of an efficient workflow (Figure 1), whose software tools allowed us to reduce time and effort. The freely available modeling software may provide a marked contribution to create subject-specific models and simulations of movement more efficiently, saving time and effort, and without necessarily requiring high skilled expertise.

In summary, our study revealed that the uncertainties in parameter identification of subject-specific musculoskeletal models have a moderate effect on model predictions, and there are not specific parameters considered crucial for the degree of model robustness. However, the precision of model predictions should be considered carefully with regards to the intended application. In fact, model predictions such as joint contact forces may present maximum ranges of variability that are in the same order of magnitude of their values.

## Supporting Information

Appendix S1 Musculoskeletal modeling software.
(PDF)
