# Supplementary material for: Are Subject-Specific Musculoskeletal Models Robust to the Uncertainties in Parameter Identification?
Source: PLoS One. 2014 Nov 12;9(11):e112625. doi: 10.1371/journal.pone.0112625 (PMC4229232; doi:10.1371/journal.pone.0112625)
Supplement: Appendix S1 — Musculoskeletal modeling software. (PDF) [file pone.0112625.s001.pdf]

Appendix S1 Musculoskeletal Modeling Software

The software system for subject-specific musculoskeletal modeling is a result of the NMS Physiome project[1]. The developed tools, NMSBuilder and the Probabilistic Musculoskeletal Modeling module (PMM), are freely available and represent a complete workflow for probabilistic modeling of the musculoskeletal system. Both tools leverage OpenSim for multibody modeling and simulation of movement. Specifically, they use the OpenSim application programming interface (API), a set of libraries that provides access to OpenSim classes and objects for batch processing [1], interfacing external programs to the OpenSim source code. Software and additional resources including manuals and videos can be downloaded from the dedicated SimTK.org project page[2].

NMSBuilder

NMSBuilder is an application with a graphical user interface (GUI) that integrates the functionalities of the Multimod Application Framework (MAF) [2] and the OpenSim 3.0 API. NMSBuilder provides two major features: biomedical data processing, and creation of subject-specific musculoskeletal models and simulations leveraging OpenSim. In summary, databases of disparate biomedical data can be created and organized into a hierarchical structure, where each data block, named Virtual Medical Entity (VME), contains a dataset, a pose matrix defining location and orientation of the dataset, and associated metadata attributes. The data can be explored using the available views that are interactive and provide the proper visualization of each VME type. Operations are applied to VMEs to modify them or generate new ones. For example, it is possible to create service entities (e.g., anatomical landmarks, reference systems, parametric solids), perform procedural manipulation (e.g., create distance meters, create wrapped action lines, extract isosurfaces), modify datasets (e.g., fuse, move, scale VMEs, deform surfaces, crop volumes). The musculoskeletal modeling features allow to convert MAF-based data structures into the OpenSim 3.0 model file format, and create and launch the scripts to configure and run OpenSim simulations. This relies on the creation of specialized components in NMSBuilder, allowing to represent an OpenSim model as C++ commands of the OpenSim API, edit the model, compile the C++ commands to generate the model, define the simulation to be run. The creation of an OpenSim model can be performed using a wizard (i.e., a sequence of user-friendly dialog boxes in NMSBuilder) hiding the backend creation of C++ API commands. These functionalities create the

[1] http://www.nmsphysiome.eu/

[2] https://simtk.org

basic components of a multibody OpenSim model: definition of rigid bodies (calculation of inertial properties), idealized joints (reference frames and kinematic constraints) and musculotendon actuators (geometry and parameters). The software development was performed using the eXtreme Programming approach[3], which is a methodology for software development stressing on customer satisfaction and accounting for the fact that providers (i.e., software developers) and customers (i.e., application experts) may be physically remote.

Probabilistic Musculoskeletal Modeling module (PMM)

PMM interfaces MATLAB® (The MathWorks, Inc., USA) and the OpenSim API, providing a simple GUI written in MATLAB to access, visualize and modify OpenSim model variables, and repeatedly run simulations of movement. The user can import any OpenSim musculoskeletal model, choose which variables have a probabilistic distribution, select the type of distribution, and assign the corresponding parameter values. Deterministic variables can also be modified. Once all variables have been defined, they can be statistically sampled according to the Latin Hypercube Sampling (LHS) strategy [3], selecting arbitrary sample sizes. The corresponding OpenSim model files are then generated using the modified variables. An additional GUI allows the user to choose which OpenSim simulations are wished to be repeatedly run using the generated models. PMM is compatible with Octave, although the GUI is currently not supported in Octave.

References

1. Seth A, Sherman M, Reinbolt JA., Delp SL (2011) OpenSim: a musculoskeletal modeling and simulation framework for in silico investigations and exchange. Procedia IUTAM 2: 212–232.
2. Viceconti M, Zannoni C, Testi D, Petrone M, Perticoni S, et al. (2007) The multimod application framework: a rapid application development tool for computer aided medicine. Computer methods and programs in biomedicine 85: 138–151.
3. McKay MD, Beckman RJ, Conover WJ (1979) Comparison of Three Methods for Selecting Values of Input Variables in the Analysis of Output from a Computer Code. Technometrics 21: 239–245.

[3] http://www.extremeprogramming.org/
